# Gender-specific effects of soybean consumption on cardiovascular events in elderly individuals from rural Northeast China – a prospective cohort study

**DOI:** 10.1186/s12877-023-04209-1

**Published:** 2023-08-23

**Authors:** Shasha Yu, Hongmei Yang, Bo Wang, Xiaofan Guo, GuangXiao Li, Yingxian Sun

**Affiliations:** 1 Department of Cardiology, First Hospital of China Medical University, 155 Nanjing North Street, Heping District, Shenyang, 110001 China; 2 Department of Clinical Epidemiology, Institute of Cardiovascular Diseases, First Hospital of China Medical University, Shenyang, 110001 China

**Keywords:** Soybean, Soybean product, Cardiovascular events, Coronary heart disease, Stroke, Cardiovascular mortality, All-cause mortality

## Abstract

**Background:**

The impact of consuming soybean and its products on cardiovascular events (CVEs), cardiovascular mortality, and all-cause mortality remains unclear. This study aimed to examine the prospective association of soybean consumption with CVEs, cardiovascular mortality, and all-cause mortality among the elderly population in rural China.

**Methods:**

The Northeast China Rural Cardiovascular Health Study included 2477 elderly individuals (mean age 67 ± 6 years, 49.97% men) in the initial phase of the study from 2012 to 2013, with a follow-up period between 2015 and 2017. Soybean consumption was categorized as follows: low-frequency consumption: rare consumption; moderate-frequency consumption: two to three times/week; high-frequency consumption: ≥ four times/week. Cox proportional hazard analysis assessed the potential relationship of soybean consumption with CVEs, cardiovascular mortality, and all-cause mortality.

**Results:**

The prevalence of soybean and its product consumption was as follows: 38.3% for low-frequency consumption (43.8% for women; 32.8% for men), 49.7% for moderate-frequency consumption (45.8% for women; 53.7% for men), and 11.9% for high-frequency consumption (10.4% for women; 13.5% for men). After adjusting for possible confounders, Cox proportional hazard analysis revealed that the frequency of soybean consumption was an effective predictor of CVEs [Hazard ratio (HR) _high_ (95% CI): 0.555 (0.348, 0.883)], stroke [HR _moderate_ (95% CI): 0.672 (0.494, 0.913); HR _high_ (95% CI): 0.483 (0.276, 0.842)], and all-cause mortality [HR _high_ (95% CI): 0.540 (0.310, 0.942)] in the overall older population. High-frequency consumption of soybean [HR (95% CI): 0.467 (0.225, 0.968)] and moderate-frequency consumption [HR (95% CI): 0.458 (0.270, 0.779)] were associated with stroke events in older men and women, respectively. In addition, high-frequency consumption of soybean [HR (95% CI): 0.437 (0.197, 0.968)] decreased the risk of CVEs in older women.

**Conclusion:**

Soybean consumption is closely associated with CVEs and all-cause mortality in older individuals residing in rural areas, with a significant gender discrepancy in this relationship. These findings provide new insights into the impact of soybean consumption on cardiovascular well-being in the elderly rural population, thus enhancing our understanding of this field of interest.

## Background

Previous studies have highlighted the crucial role of diet in the progression of chronic diseases [1, 2]. In Asian countries, soybeans and their products, like Tofu, soybean milk, and Tofu jelly, are widely consumed, especially in China. Soybeans and their products have demonstrated numerous beneficial effects on chronic diseases, partly attributed to their ability to lower serum lipid profiles. Non-nutritive substances, such as soy isoflavones, have been associated with preventing and treating different chronic diseases. Moreover, soy dietary fiber has been recognized for its health-promoting properties [3]. Furthermore, soybeans and soy foods improve vascular health, preserve bone mineral density, benefit renal function, and reduce menopausal symptoms [4]. Based on meta-analyzes and recent clinical trials, soy isoflavones have been found to effectively reduce hot flashes, improve the quality of life, and lower the cardiovascular risk in women [5]. Therefore, in recent years, nutritionists have suggested replacing animal-based foods with soybean foods to harness their nutritional benefits.

In recent years, China has been facing the impact of an “aging tsunami,” which has emerged as a significant concern due to the increasing proportion of older individuals in the general population [6]. According to the data from the Seventh National Population Census in 2020, the proportion of individuals aged > 60 and > 65 years has increased by 5.44% (from 13.26% to 2010 to 18.70% in 2020) and 4.6% (from 8.9 to 13.5%) over the past decade, respectively [7]. In rural China, the proportion of individuals aged ≥ 60 and ≥ 65 years is 23.8% and 17.7%; these are 7.99% and 6.61% higher than those in urban areas, respectively. Data from the Seventh National Population Census highlights the need to address the challenges of aging in rural areas. Chronic diseases, particularly cardiovascular diseases (CVDs), are significantly frequent among the aging population, necessitating effective strategies to reduce the incidence and mortality of CVDs in this demographic.

Several prospective cohort studies and meta-analyses have reported that a higher-frequency consumption of soybeans and their products is associated with a lower mortality risk and CVD [8]. However, the association between soybean consumption, all-cause mortality, and CVD remains unclear. Meta-analyses have reported conflicting results, with one showing no association and two others showing a positive association [9–11]. Previous studies were predominantly conducted in urban or developed areas, with limited information from the rural regions. Moreover, these studies typically recruited participants with inadequate ability to provide accurate information for devising the prevention strategy for older individuals. This study investigated the potential association of the frequency of consuming soybean and its products with cardiovascular events (CVEs), cardiovascular mortality, and all-cause mortality among rural older individuals.

## Method and materials

### Study design and participants

The Northeast China Rural Cardiovascular Health Study (NCRCHS) is a community-based prospective cohort study conducted in rural areas of Northeast China. Detailed information regarding the specific sampling methods and admission criteria has been previously described [12]. The Ethics Committee of China Medical University approved the present study (Shenyang, China AF-SDP-07-1, 0–01). The baseline data for this study were obtained from the 2012‒2013 survey with 3128 participants (aged > 60 years). Among them, 446 participants were excluded from the analysis of predictors for new-onset CVEs due to a history of stroke, myocardial infarction (MI), or severe congestive heart failure (CHF). Furthermore, 205 participants were excluded because of missing important information. Finally, 2477 participants were enrolled, and their data were carefully collected. The cohort was followed up from 2015 to 2017.

### Baseline data

The history of stroke, coronary heart disease (CHD), and heart failure was defined at study initiation based on self-reporting and confirmation through medical records. Weight and height measurements were taken with participants wearing light clothing and no shoes. Waist circumference was measured at the umbilicus using non-elastic tape. Body mass index (BMI) was computed as weight in kilograms divided by the square of height in meters. Blood pressure (BP) was measured thrice with participants seated after a minimum of 5 min of rest, using a standardized automatic electronic sphygmomanometer (HEM-907; Omron, Tokyo, Japan). Fasting blood samples were collected in the morning from participants who had fasted for at least 12 h. The enzymatic analysis helped measure fasting plasma glucose (FPG), low-density lipoprotein cholesterol (LDL-C), and other routine blood biochemical indexes. Physical activity, which included occupational and leisure-time physical activity, was categorized into three classes: low, moderate, and heavy [13]. Annual income was categorized into ≤ 5000 CNY/year, 5000–20,000 CNY/year, and > 20,000 CNY/year. Sleep duration was categorized into four groups: ≤7 h/day, 7–8 h/day, 8–9 h/day, and > 9 h/day. The questionnaire also requested details on the typical weekly consumption of various foods, with intake measured in grams per week. The following scale was used to evaluate vegetable consumption: rarely = 3, < 1000 g = 2, > 1000 g = 1, and > 2000 g = 0. Meat consumption, including red meat, fish, and poultry, was evaluated using the following scale: rarely = 0, < 250 g = 1, > 250 g = 2, and > 500 g = 3. Each participant was assigned a distinct diet score (meat consumption score plus vegetable consumption score, ranging from 0 to 6). Lower diet scores indicated adherence to the Chinese diet, while higher scores reflecting a higher meat intake and lower vegetable intake showed greater compliance with a Westernized diet. The “ATTICA study” also utilized identical formulas for diet calculation [14]. The consumption of soybean and its products was divided into three groups: low-frequency consumption: rare consumption, moderate-frequency consumption: two to three times per week, and high-frequency consumption: ≥ four times per week.

### Follow-up

The median follow-up period was 4.66 years. Incident CVEs were defined as new-onset strokes or CHDs during the follow-up period. The occurrences of CHD and stroke were explicitly identified. All clinical data, including medical records and death certificates, were collected for individuals who reported potential diagnoses or deaths. An independent end-point evaluation committee reviewed and made decisions on all submissions. The stroke cases were defined according to the WHO Multinational Monitoring of Trends and Determinants in Cardiovascular Disease (MONICA) criteria [15, 16], which consider rapidly developing signs of focal or global disturbance of cerebral function, lasting more than 24 h (unless interrupted by surgery or death) with no apparent non-vascular causes. Ischemic stroke was characterized as a stroke diagnosed with either thrombosis or embolism; in contrast, hemorrhagic stroke was described as a case of stroke with a subarachnoid or intracerebral hemorrhage diagnosis. Transient ischemic attacks and chronic cerebral vascular diseases were excluded. The diagnosis of CHD encompassed any revascularization operation, hospitalized angina, hospitalized myocardial infarction, or CHD mortality [17]. Furthermore, all-cause and CVD mortality were included in our study. Confirmation of deaths was done through hospital records and direct contact with their families. Death from CVD was confirmed based on autopsy reports, death certificates, medical record abstracts, or information obtained from family members [18]. The end-point assessment committee, consisting of certified neurologists, cardiologists, and others, independently reviewed all records.

### Statistical analysis

Descriptive statistics were calculated for continuous (reported as mean values and standard deviations) and categorical variables (reported as numbers and percentages). Differences among categories were evaluated using appropriate tests, such as t-test, ANOVA, non-parametric tests, or the χ2-test. Cox proportional hazards model calculated the hazard ratios for CVEs, cardiovascular mortality, and all-cause mortality. All statistical analyses were performed using SPSS version 20.0 software (SPSS Inc., Chicago, Illinois, USA). Statistical significance was set at P < 0.05.

## Results

Table 1 shows the baseline characteristics of the study participants according to gender. In this study, male participants were significantly older than females. The prevalence of the Han race and current smoking and drinking habits were higher in men. In contrast, the proportions of those married, with primary or lower education, shorter sleep duration, and lower annual income, were lower in men than women. At baseline, male participants exhibited significantly higher diastolic BP (DBP), estimated glomerular filtration rate (eGFR), and uric acid values. Meanwhile, BMI, TC, TG, and FPG were relatively higher in the women. Furthermore, during the follow-up period, male participants had more elevated systolic BP (SBP), DBP, BMI, and FPG values, while female participants had higher values of HDL-C and TG.

**Table 1:** Characteristics of study participants according to gender

Baseline characteristics	Total (n = 2477)	Male (n = 1238)	Female (n = 1239)	P-value
Age, mean (SD), years	66.80 ± 5.71	67.10 ± 5.88	66.50 ± 5.51	0.009
Race (Han), n (%)	2369(95.6)	1193(96.4)	1176(94.9)	0.047
Current smoking (yes), n (%)	920(37.1)	622(50.2)	298(24.1)	< 0.001
Current drinking (yes), n (%)	545(22.0)	489(39.5)	56(4.5)	< 0.001
Marriage status (yes), n (%)	2456(99.2)	1219(98.5)	1237(99.8)	< 0.001
Primary and below education, n (%)	1860(75.1)	799(64.5)	1061(85.6)	< 0.001
Sleep duration, n (%)				< 0.001
≤7 h/day	1363(55.2)	617(50.0)	746(60.4)	
7-8 h/day	584(23.6)	306(24.8)	278(22.5)	
8-9 h/day	352(14.3)	210(17.0)	142(11.5)	
>9 h/day	171(6.9)	102(8.3)	69(5.6)	
Annual income (CNY/year), n (%)				0.421
≤5000	575(23.2)	281(22.7)	294(23.8)	
5000–20,000	1385(56.0)	686(55.5)	699(56.5)	
>20,000	514(20.8)	270(21.8)	244(19.7)	
Physical activity, n (%)				< 0.001
Low	1405(57.3)	614(50.0)	791(64.6)	
Moderate	404(16.5)	203(16.5)	201(16.4)	
severe	643(26.2)	410(33.4)	233(19.0)	
Die score (≥ 3)	1021(41.3)	615(49.8)	406(32.8)	< 0.001
SBP, mean (SD), mmHg	151.59 ± 23.94	152.10 ± 23.26	151.08 ± 24.60	0.294
DBP, mean (SD), mmHg	82.01 ± 11.68	83.41 ± 11.35	80.61 ± 11.84	< 0.001
BMI, mean (SD), kg/m2	24.31 ± 3.66	24.10 ± 3.38	24.51 ± 3.90	0.005
TC, mean (SD), mmol/L	5.43 ± 1.09	5.16 ± 1.02	5.70 ± 1.09	< 0.001
HDL-C, mean (SD), mmol/L	1.44 ± 0.41	1.44 ± 0.47	1.44 ± 0.37	0.815
LDL-C, mean (SD), mmol/L				
TG, mean (SD), mmol/L	1.26(0.92,1.93)	1.11(0.82, 1.67)	1.47(1.05, 2.16)	< 0.001
FPG, mean (SD), mmol/L	6.06 ± 1.80	5.97 ± 1.60	6.15 ± 1.97	0.016
eGFR, mean (SD), ml/min per 1.73m^2^	83.34 ± 13.63	84.97 ± 11.92	81.72 ± 14.98	< 0.001
Uric acid, mean (SD), µmoI/L	292.52 ± 80.19	319.95 ± 80.19	265.18 ± 69.55	< 0.001
**Follow-up Characteristics**				
SBP, mean (SD), mmHg	135.99 ± 21.60	139.14 ± 21.34	132.10 ± 21.29	< 0.001
DBP, mean (SD), mmHg	80.01 ± 11.61	81.85 ± 11.68	77.74 ± 11.12	< 0.001
BMI, mean (SD), kg/m2	23.64 ± 3.55	24.28 ± 3.28	22.86 ± 3.72	< 0.001
HDL-C, mean (SD), mmol/L	1.46 ± 0.38	1.44 ± 0.39	1.48 ± 0.37	0.005
TG, mean (SD), mmol/L	1.39(0.97,2.09)	1.22(0.86,1.80)	1.64(1.11, 2.35)	< 0.001
FPG, mean (SD), mmol/L	5.60 ± 1.28	5.75 ± 1.48	5.41 ± 0.95	< 0.001
Metabolic syndrome, n (%)	1188(24.0)	572(22.2)	616(25.8)	0.203

SD Standard deviation, CNY Chinese Yuan, SBP Systolic blood pressure, DBP Diastolic blood pressure, BMI Body mass index, HDL-C High-density lipoprotein cholesterol, TG Triglycerides, FPG Fasting plasma glucose

Table 2 presents the characteristics of participants based on their soybean consumption. As the frequency of soybean consumption increased among female participants, the current smoking rate increased simultaneously. Similarly, the primary or lower educational status rate increased with higher soybean consumption frequency. The annual income distribution varied among male and female participants, depending on the frequency of soybean consumption. Participants with low-frequency soybean consumption had a significantly higher rate of annual income at ≤ 5000 CNY/year. Among the women, there were more participants with low physical activity in the ≥ four times/week consumption-frequency group than in the other two groups. The mean values of BMI at baseline and follow-up increased as the frequency of soybean consumption increased among female participants.

**Table 2:** Characteristics of study participants according to soybean and soybean product consumption

Baseline characteristics	Low frequency consumption (n = 946)		Moderate frequency consumption (n = 1232)		High frequency consumption (n = 296)	
	Male (n = 406)	Female (n = 543)	Male (n = 665)	Female (n = 567)	Male (n = 167)	Female (n = 129)
Age, mean (SD), years	67.17 ± 6.02	66.44 ± 5.30	67.19 ± 5.96	66.53 ± 5.60	66.58 ± 5.22	66.63 ± 5.98
Race (Han), n (%)	391(96.3)	521(95.9)	643(96.7)	536(94.5)	159(95.2)	119(92.2)
Current smoking (yes), n (%)	210(51.7)	**155(28.5)**	327(49.2)	**118(20.8)**	85(50.9)	**25(19.4)**
Current drinking (yes), n (%)	143(35.2)	24(4.4)	273(41.1)	24(4.2)	73(43.7)	8(6.2)
Marriage status (yes), n (%)	400(98.5)	542(99.8)	655(98.5)	566(99.8)	164(98.5)	129(100)
Primary and below education, n (%)	271(66.7)	**465(85.6)**	424(63.8)	**485(85.5)**	104(62.3)	**111(86.0)**
Sleep duration, n (%)						
≤7 h/day	211(52.1)	321(59.3)	326(49.1)	355(62.8)	80(48.2)	70(54.3)
7-8 h/day	87(21.5)	126(23.3)	176(26.5)	126(22.3)	43(25.9)	26(20.2)
8-9 h/day	77(19.0)	59(10.9)	108(16.3)	60(10.6)	25(15.1)	23(17.8)
>9 h/day	30(7.4)	35(6.5)	54(8.1)	24(4.2)	18(10.8)	10(7.8)
Annual income (CNY/year), n (%)						
≤5000	**114(28.1)**	**163(30.1)**	**144(21.7)**	**114(20.1)**	**23(13.9)**	**17(13.2)**
5000–20,000	**223(54.9)**	**296(54.7)**	**373(56.1)**	**328(57.8)**	**90(54.2)**	**75(58.1)**
>20,000	**69(17.0)**	**82(15.2)**	**148(22.3)**	**125(22.0)**	**53(31.9)**	**37(28.7)**
Physical activity, n (%)						
Low	178(44.5)	**308(57.2)**	352(53.3)	**389(69.6)**	84(50.6)	**94(73.4)**
Moderate	68(17.0)	**114(21.2)**	105(15.9)	**73(13.1)**	30(18.1)	**14(10.9)**
severe	154(38.5)	**116(21.6)**	204(30.9)	**97(17.4)**	52(31.3)	**20(15.6)**
Die score (≥ 3)	**174(42.9)**	**141(26.0)**	**346(52.1)**	**214(37.7)**	**95(57.2)**	**51(39.5)**
SBP, mean (SD), mmHg	150.93 ± 24.15	150.45 ± 24.46	152.31 ± 23.13	151.27 ± 25.05	154.13 ± 21.41	152.93 ± 23.23
DBP, mean (SD), mmHg	82.98 ± 11.37	80.04 ± 12.16	83.47 ± 11.26	80.96 ± 11.65	84.26 ± 11.72	81.52 ± 11.24
BMI, mean (SD), kg/m2	23.80 ± 3.70	**24.13 ± 4.00**	24.19 ± 3.14	**24.82 ± 3.78**	24.48 ± 3.45	**24.84 ± 3.88**
TC, mean (SD), mmol/L	5.08 ± 1.07	5.65 ± 1.05	5.18 ± 1.00	5.73 ± 1.11	5.28 ± 0.96	5.77 ± 1.10
HDL-C, mean (SD), mmol/L	1.43 ± 0.45	1.45 ± 0.35	1.45 ± 0.45	1.44 ± 0.37	1.43 ± 0.42	1.46 ± 0.43
LDL-C, mean (SD), mmol/L	2.86 ± 0.86	3.19 ± 0.83	2.88 ± 0.76	3.32 ± 0.85	3.05 ± 0.77	3.41 ± 0.91
TG, mean (25%, 75%), mmol/L	1.08(0.80, 1.55)	1.44(1.07, 2.18)	1.11(0.82, 1.69)	1.52(1.04, 2.15)	1.13(0.85, 2.06)	1.44(1.04, 2.10)
FPG, mean (SD), mmol/L	5.86 ± 1.66	6.13 ± 2.09	6.01 ± 1.50	6.16 ± 1.91	6.10 ± 1.84	6.18 ± 1.76
eGFR, mean (SD), ml/min per 1.73m^2^	84.98 ± 12.66	82.13 ± 15.52	85.03 ± 11.27	80.97 ± 14.83	84.66 ± 12.60	83.29 ± 13.18
Uric acid, mean (SD), µmoI/L	322.62 ± 87.20	266.75 ± 66.79	317.99 ± 77.41	264.84 ± 72.89	321.24 ± 78.10	260.15 ± 65.93
**Follow-up Characteristics**						
SBP, mean (SD), mmHg	148.92 ± 22.59	145.75 ± 25.08	145.98 ± 21.77	146.00 ± 24.19	144.05 ± 19.65	146.27 ± 22.15
DBP, mean (SD), mmHg	82.51 ± 11.63	78.96 ± 12.38	82.04 ± 11.66	80.17 ± 12.25	81.34 ± 11.83	80.32 ± 11.43
BMI, mean (SD), kg/m2	24.51 ± 3.59	**23.10 ± 3.96**	24.64 ± 3.71	**23.81 ± 4.17**	25.19 ± 4.12	**23.74 ± 4.29**
TC, mean (SD), mmol/L	4.84 ± 0.90	5.19 ± 0.94	4.76 ± 0.93	5.32 ± 0.97	4.95 ± 0.93	5.32 ± 0.97
LDL-C, mean (SD), mmol/L	3.08 ± 0.83	3.30 ± 0.85	3.02 ± 0.81	3.44 ± 0.88	3.16 ± 0.78	3.37 ± 0.97
HDL-C, mean (SD), mmol/L	1.38 ± 0.40	**1.38 ± 0.41**	1.36 ± 0.40	**1.31 ± 0.37**	1.35 ± 0.39	**1.34 ± 0.40**
TG, mean (25%, 75%), mmol/L	1.22(0.89, 1.81)	1.44(1.07, 2.18)	1.17(0.84, 2.70)	1.52(1.04, 2.15)	1.37(0.91, 1.91)	1.44(1.04, 2.10)
FPG, mean (SD), mmol/L	5.98 ± 1.98	5.97 ± 1.45	6.00 ± 1.94	5.99 ± 1.63	6.13 ± 1.45	6.07 ± 1.61
eGFR, mean (SD), ml/min per 1.73m^2^	84.04 ± 12.08	83.34 ± 12.74	84.48 ± 12.36	82.83 ± 13.18	81.88 ± 12.69	83.34 ± 11.27
Uric acid, mean (SD), µmoI/L	317.11 ± 78.08	263.75 ± 65.33	320.55 ± 78.99	269.81 ± 67.07	334.29 ± 77.48	270.93 ± 69.97

SD Standard deviation; Bold type means *P* < 0.05; CNY Chinese Yuan, SBP Systolic blood pressure, DBP Diastolic blood pressure, BMI Body mass index, HDL-C High-density lipoprotein cholesterol, TG Triglycerides, FPG Fasting plasma glucose

**Fig. 1:**
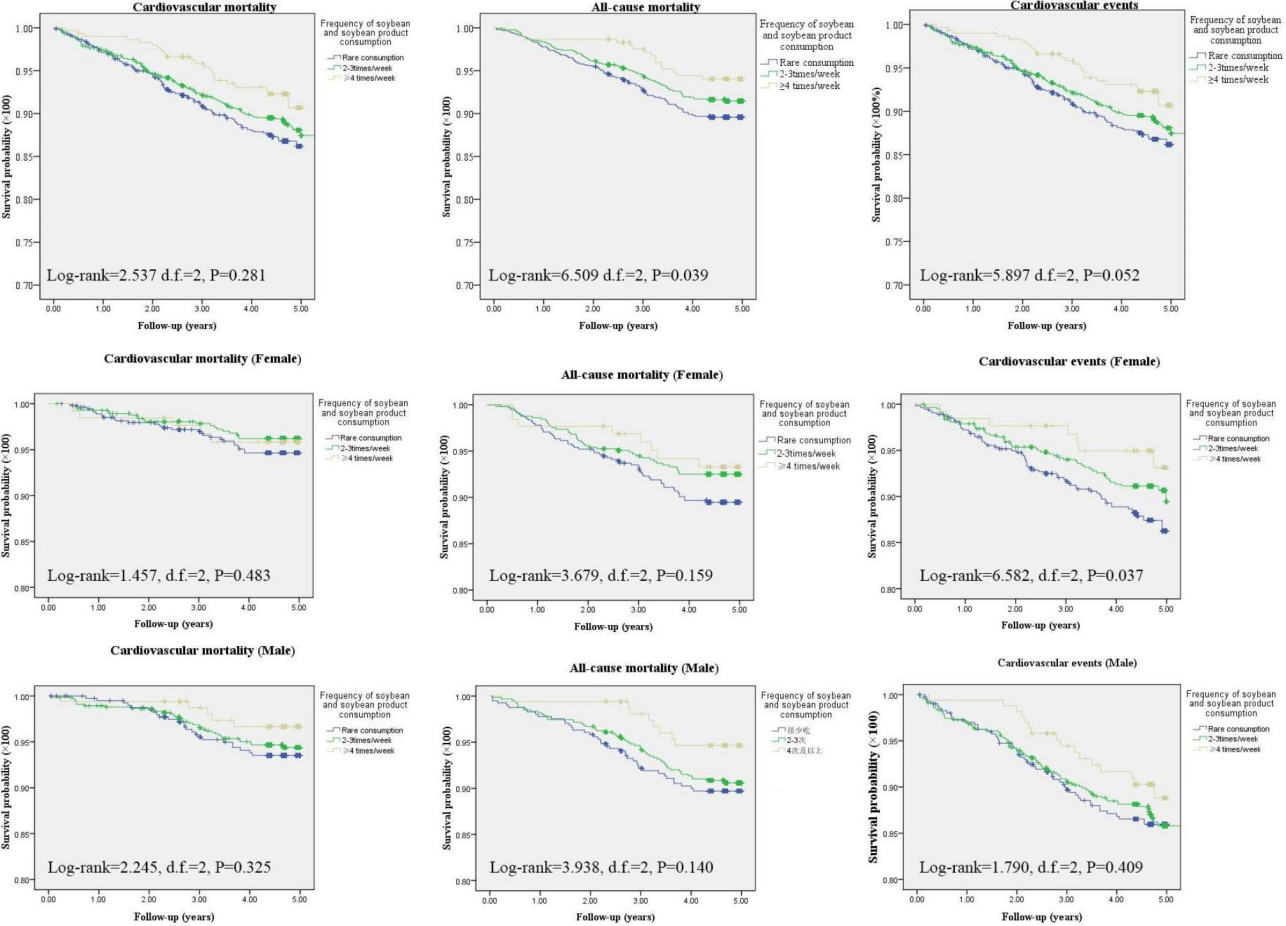
Cardiovascular events, cardiovascular and all-cause mortality in the three groups according to the frequency of soybean and soybean product consumption

Figure 1 illustrates the Kaplan-Meier curve analyzing the association between soybean consumption, CVEs, cardiovascular mortality, and all-cause mortality. The data revealed a significant correlation between soybean intake and its products, and the all-cause mortality in the study population. Moreover, soybean consumption was associated with CVEs in women.

**Fig. 2:**
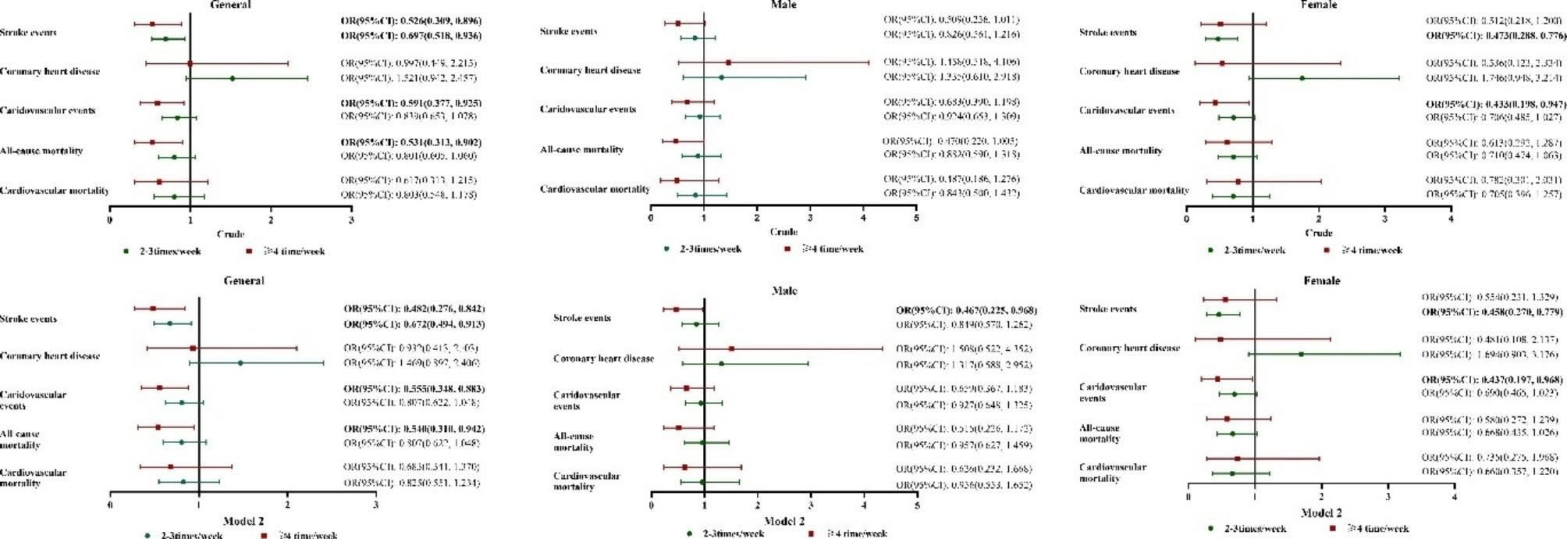
Crude did not adjust for any possible confounders. Model 1 adjusted for age, gender, race, educational status, sleep duration, annual income, current smoking, current drinking, physical activity, baseline systolic blood pressure, diastolic blood pressure, body mass index, low density lipoprotein cholesterol, fasting plasma glucose, eGFR, total cholesterol, triglyceride, uric acid, and diet score relative hazard ratio and 95% confidence intervals (CI) of cardiovascular events, cardiovascular and all-cause mortality in participants divided into three groups according to the frequency of soybean consumption. Rare consumption of soybean and soybean product represent the reference group

Figure 2 displays the Cox regression analysis examining the potential predictive effect of soybean consumption on CVEs, cardiovascular mortality, and all-cause mortality. After adjusting for possible confounders, a higher frequency of soybean consumption (HR _moderate_: 0.672; HR _high_: 0.484) was associated with stroke events in the general population. Furthermore, high-frequency consumption of soybean was an effective predictor of CVEs [HR (95% CI): 0.555 (0.348, 0.883)] and all-cause mortality [HR (95% CI): 0.540 (0.310, 0.942)]. In older male individuals, high-frequency consumption of soybean [HR (95% CI): 0.467 (0.225, 0.968)] and moderate-frequency consumption of soybean [HR (95% CI): 0.458 (0.270, 0.779)] were significant predictors of stroke in male and female older individuals, respectively. Soybean consumption was significantly associated with CVEs [HR _high_: 0.437] only among female participants.

## Discussion

In this prospective study from rural Northeast China, we found that higher intakes of soybean and its products were associated with reduced risk of stroke events, CVEs, and all-cause mortality in the older population. Moderate and high-frequency soybean consumption correlated with fewer stroke cases in older women and men, respectively. The frequency of consuming soybean and its products was associated with CVEs only among females.

Previous research has also examined the association between soybean consumption and all-cause mortality. Mariko Nakamoto et al. demonstrated that a higher intake of soy products might decrease the risk of all-cause mortality, especially in middle-aged Japanese individuals [9]. They attributed this protective effect to the constituent, isoflavones, which exert hormone-independent activities, such as proapoptotic, anti-oxidative, anti-inflammatory, and anti-angiogenic effects [9]. However, not all previous studies have reached a unified conclusion on the impact of soy products and isoflavones on all-cause mortality. Ponzo V et al. reported a positive association between a high total intake of isoflavones and high all-cause mortality, conflicting with other similar studies [19–21]. These studies reported the inverse association between soybean consumption and all-cause mortality, prominent in Asian countries like Japan, Korea, and China [9, 22]. This discrepancy might be attributed to the different dietary habits in Asian countries, where the intake of isoflavones in Asian countries (> 30 mg/day) was significantly higher than in Western countries (< 1 mg/day) [23, 24]. A nationwide prospective cohort study conducted in China illustrated that those participants with higher daily soy consumption levels (> 60 g/day) had higher rates of CVD-free and overall survival compared to those consuming < 15 g/day [25]. Our study contributes novel evidence regarding the protective effect of soybean consumption on all-cause mortality in the rural Chinese population. We showed that an increased frequency of consuming soybean and its products reduced the risk of all-cause mortality, specifically in older Chinese individuals, over a medium follow-up period of 4.6 years. This beneficial effect might be attributed to the coexistence of other socioeconomic factors and healthier lifestyles in older individuals, such as a relatively lower prevalence of current smoking and higher annual income. Furthermore, the antioxidant and anti-inflammatory effects of soybeans might also help to prevent all-cause mortality by inhibiting the generation of reactive oxygen species, lipid peroxidation, and protein oxidation [26].

Except for all-cause mortality, a higher frequency of soybean consumption was also associated with CVEs. Previous studies have confirmed the association between soybean consumption and various health benefits in reducing chronic diseases, such as obesity, CVD, insulin resistance/type II diabetes, and immune disorders. The chemical components responsible for these effects are the soy bioactive peptides, which possess hypolipidemic, anti-hypertensive, anti-cancer, anti-inflammatory, antioxidant, and immunomodulatory properties [27]. We further conducted a stratified analysis by gender. Although we found no significant association between soybean consumption and CVEs in men, there was a significant reduction in the risk of CVEs among older female individuals with a higher frequency of soybean consumption. This gender-specific association between soybean consumption and cardiometabolic health, with beneficial effects in women and unfavorable impact in men, has been reported in previous studies [28, 29]. This gender discrepancy could be attributed to another mechanism of action for the effects of soybean on CVEs, namely the antiestrogen-like effect. Since the present study enrolled older individuals, the female participants were in a postmenopausal state where the antiestrogen-like effect plays a more significant role than in men. A cohort study of 40,462 Japanese people with an average follow-up of 12.5 years found that total soy intake was linked to fewer cerebral and myocardial infarctions in postmenopausal women and not in men or premenopausal women [30].

Many previous studies have suggested the beneficial effects of soybean and its isoflavones in correcting lipid metabolism changes in postmenopausal women, which might favor preventing CVEs [31]. Meta-analyses have shown that isoflavones from soybean effectively reduce hot flashes, improve the quality of life, lower cardiovascular risk by optimizing lipid profile, and improve bone mineral density and other markers of bone resorption [5]. These beneficial effects of soybean support the favorable impact of regular soybean consumption on the health and quality of life of peri- and postmenopausal women [32].

A remarkable finding is the correlation of soybean consumption with stroke events and the absence of such correlation with CHD in the general population. A gender-based stratification analysis revealed that the significant association between soybean consumption and stroke events persisted in older individuals, irrespective of gender. However, inconsistent results were observed in studies analyzing the association between soybean consumption and the risk of CHD [33–35]. Danxia Yu et al. reported a positive and dose-dependent association between soy intake and the risk of CHD [35]. A high habitual soy intake was hypothesized to have adverse effects on the development of CHD in men, partly due to the elevated IL-8 and PAI-1 levels among those with high soy intake [35]. In contrast, soy food consumption was significantly and inversely associated with the risk of CHD among Chinese women [36]. Similarly, Massimiliano Ruscica et al. claimed that the intake of whole soy foods (corresponding to 30 g/day of protein) had significantly impacted cardiovascular risk [37]. A prospective cohort study in Korea also inferred that whole soy foods were associated with a decreased risk of CVD in premenopausal women [38]. Another research conducted among 64,915 Chinese women with a mean follow-up of 2.5 years demonstrated that participants who consumed ≥ 11.19 g/day total soy protein had a significantly lower risk of CHD than those consuming < 4.5 g/day [39].

The inconsistency in these findings might be attributed to using various soy products, different intake levels of soy foods, and variations in dietary control [40]. The difference in the association of soybean consumption with CHD and stroke could be attributed to the variations in genetic susceptibility, similar to the increased likelihood of hypertension to induce stroke instead of CHD in the Asian population [41]. The beneficial effects of soybean consumption might be more pronounced in stroke cases. Do Yeon Jeong et al. determined that *Chungkookjang*, a traditional Korean fermented soybean food, possesses anti-stroke, anti-diabetic, and thrombolytic properties; it reduced the susceptivity to injury from ischemic stroke by improving the gut microbiome, increasing blood flow to the brain, and suppressing systemic inflammation [42]. Hiromitsu Watanabe’s study results suggested that Japanese soybean paste (Miso) might have protective effects against stroke despite its high salt content [43]. Consistent with these findings, our study revealed that higher frequencies of soybean and its product intake were associated with stroke events, even when stratified by gender. In addition to the higher frequency of soybean consumption, a case-control study conducted in Southern China found that a relatively higher soy food intake was negatively associated with ischemic stroke [odds ratio (OR) _for 50–299 g/week_: 0.63; OR for _≥ 300 g/week_: 0.23] [44]. Ting Xue et al. also discovered that consuming ≥ 60 g/day of soy was associated with a reduced risk of non-fatal stroke compared to consuming < 15 g/day [45].

However, a meta-analysis of observational studies concluded that no association was found between soy intake and the risk of stroke or CHD when cohort studies were combined. Nevertheless, case-control studies showed a significant inverse association between soy intake and the risk of stroke and CHD. Therefore, future studies using prospective designs with validated questionnaires and controlling for important confounders are warranted.

There are some limitations in the current study. Firstly, we only collected dietary intake information at baseline, which can undergo fluctuations and be influenced by various factors as people grow older, as suggested by previous studies [37]. Secondly, our study included participants > 60 years. Therefore, when stratifying the population by gender, we could not perform Cox proportional hazards regression analysis after stratification based on menopausal status. Thirdly, there may be other potential confounders that were not comprehensively eliminated, despite controlling for various potentially essential confounders in the analyses. Finally, our study categorized participants based on the frequency of soybean consumption instead of the amount of soybean consumption; this might introduce bias that affects the relationship between soybean consumption, CVEs, cardiovascular mortality, and all-cause mortality among the elderly population in rural China.

## Conclusion

In conclusion, our results suggest the possible effects of consuming soybean and its products in decreasing the risk of all-cause mortality and CVEs among older rural individuals. Gender discrepancy existed in the relationship between soybean consumption, CVEs, and all-cause mortality. Extensive prospective studies should be conducted to investigate the optimal amount of soybean products that can benefit all-cause mortality and CVEs.

## Data Availability

The dataset supporting the conclusions of this article is available if contact correspondence author (Yingxian Sun, sunyingxiancmu1h@163.com).
